# Fabrication and In Vitro Characterization of Bioactive Glass/Nano Hydroxyapatite Reinforced Electrospun Poly(ε-Caprolactone) Composite Membranes for Guided Tissue Regeneration

**DOI:** 10.3390/bioengineering5030054

**Published:** 2018-07-15

**Authors:** Vishnu Jayakumar Sunandhakumari, Arun Kumar Vidhyadharan, Aneesh Alim, Deepan Kumar, Jayakrishnan Ravindran, Aswathy Krishna, Manoj Prasad

**Affiliations:** 1 Department of Periodontics and Oral Implantology, PMS College of Dental Science and Research Centre, Trivandrum 695028, India; 2 Department of Endodontics and Implant Dentistry, S.U.T.A.M.S Medical College, Trivandrum 695028, India; drarun_70@yahoo.com; 3 Department of Orthodontics and Dentofacial Orthopedics, PMS College of Dental Science and Research Centre, Trivandrum 695028, India; aneeshalim@gmail.com; 4 Department of Community Dentistry, PMS College of Dental Science and Research Centre, Trivandrum 695028, India; deepucv08@gmail.com; 5 Department of Oral and Maxillofacial Surgery, PSM College of Dental Science and Research, Thrissur 680519, India; drjaikishen@gmail.com; 6 Department of Oral and Maxillofacial Surgery, Amrita School of Dentistry, Cochin 682041, India; aswathyavalon@gmail.com; 7 Department of Prosthodontics, PMS College of Dental Science and Research Centre, Trivandrum 695028, India; manoj_prasad8488@yahoo.com

**Keywords:** electrospinning, guided tissue regeneration, periodontal regeneration

## Abstract

Background: Current resorbable and non-resorbable membranes act as a physical barrier to avoid connective and epithelial tissue downgrowth into the defect, favoring the regeneration of periodontal tissues. These conventional membranes possess many structural and bio-functional limitations. We hypothesized that the next-generation of guided tissue regeneration (GTR) membranes for periodontal tissue engineering will be a biologically active, spatially designed nanofibrous biomaterial that closely mimics the native extra-cellular matrix (ECM). Methods: GTR membranes made of poly(ε-Caprolactone) with a molecular weight of 80,000 reinforced with different weight concentrations of nano-Hydroxyapatite/Bioactive glass (2%, 5%, 10%, 15%) is fabricated by the method of electrospinning. After fabrication, in vitro properties are evaluated. Results: The electrospun nanofibrous membranes possessed excellent mechanical properties initially and after one month of degradation in phosphate buffer solution (PBS). Moreover, none of the fabricated membranes were found to be cytotoxic at lower concentrations and higher concentrations. Comparing the overall properties, PCL (poly(e-caprolactone)) + BG (Bioactive glass) 2% exhibited superior cell attachment and percentage of viable cells, increased fiber and pore diameter which satisfies the ideal properties needed for GTR membranes. Conclusion: Composite nanofibrous membranes prepared by electrospinning are suitable for use as a GTR membrane and are a useful prototype for further development of a final membrane for clinical use.

## 1. Introduction

Periodontitis is a chronic inflammatory disease triggered by an interaction between microbial biofilm and host response leading to gingival bleeding, connective tissue destruction, alveolar bone loss, and finally tooth loss. The desire to induce the complete regeneration of periodontal tissue has inspired the introduction of the Guided Tissue Regeneration technique [1,2,3,4,5]. The concept of guided tissue regeneration (GTR) is frequently applied in regeneration of periodontal defects, i.e., a barrier membrane is used to prevent the faster-growing connective tissue from migrating into the defect and to allow time for periodontal ligament, cementum, and bone to regenerate. The barrier membranes used for GTR can be broadly divided into three generations of membranes [6,7].

In general, the commercially available Guided tissue regeneration (GTR)/Guided bone regeneration (GBR) membranes are made of polymers, including non-degradable polytetrafluoroethylene, biodegradable polylactide (PLA), polyglycolide (PGA), polycarbonate, and collagen. Although present polymeric products show positive results in clinical studies, their weak mechanical properties and poor bone regeneration capacity are still major challenges. To overcome these problems, recent research efforts have included the incorporation of bone-like ceramics into the membranes, e.g., hydroxyapatite, tricalcium phosphate, and calcium carbonate. In these efforts, nano-sized ceramic particles are of particular interest as they mimic the mineral crystals as present in the natural tissue and have been shown to induce a significant increase in protein absorption and cell adhesion, compared to their micro-sized counterparts [8,9].

Although numerous membrane materials have been investigated, few studies have focused on the technique of membrane preparation. So far, most GTR/GBR membranes are made in the shape of porous foam, created by traditional methods such as particulate leaching, solvent casting, or gas foaming [10]. Recently, a new technique has been introduced, which is called electrospinning, and allows the preparation of thin fibrous membranes [11]. Electrospinning makes use of a high electric voltage to draw polymer solutions/melts into a whipped jet, which becomes ultrafine fibers after drying in air [12]. Fibers obtained from electrospinning are in the range of 50 nm to a few microns in diameter and generally collected in the form of a non-woven structure [13]. It has already been shown that electrospun membranes have the potential to promote osteoblastic cell function and bone regeneration [14,15]. More importantly, the pore size of the electrospun membranes in general is less than the average cell size, and previous studies have shown that such small pores do not allow cell penetration [16,17].

In view of the above, the aim of the current study was to develop a novel composite GTR membrane containing nano-Hydroxyapatite (nHA)/Bioactive glass (BG) particles and biodegradable poly(e-caprolactone) (PCL) fibers by electrospinning which ultimately helps in tissue regeneration, and to test the mechanical properties and cytotoxicity in vitro. In order to study the effect of nHA/BG, three membranes with different nHA/BG content were fabricated. Our hypothesis was that electrospinning is a suitable method to fabricate GTR membranes; and that the addition of nHA/BG improves the properties of polymeric membranes in the aforementioned terms, especially the mechanical properties and the cytotoxicity.

## 2. Materials and Methods

Laboratory facilities for the study were provided by the Biotechnology and Polymer wing of Sree Chitra Tirunal Institute for Medical Sciences and Technology, Trivandrum, India.

### 2.1. Materials

**Figure 1:**
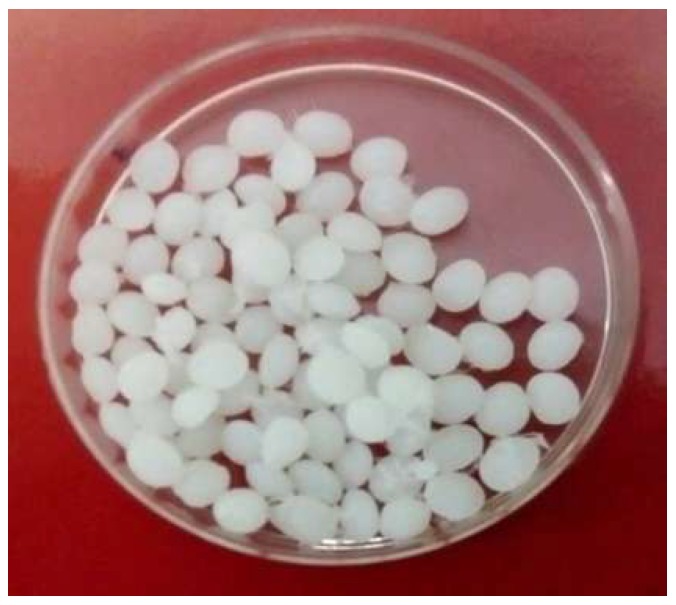
Poly(ε-Caprolactone) polymer Pellets.

The materials of the study include poly(ε-Caprolactone) polymer in the form of pellets, Bioactive glass powder, and nano-Hydroxyapatite powder. Poly(ε-Caprolactone) (PCL) pellets with an average molecular weight of Mn 80,000 was procured from Sigma Aldrich, St. Louis, MO, USA (Figure 1). The solvents used were dichloromethane and dimethylformamide (Sigma Aldrich Pt Ltd., St. Louis, MO, USA). Spray dried nano-Hydroxyapatite with average particle size of 89 nm and Wet milled Bioactive glass which is composed of 34% SiO_2_, in weight%, 4.6% MgO and 44.7% CaO, 16.2% P_2_ O_5_ and 0.5% CaF_2_ was used in this study (received from Biotechnology and Polymer wing of Sree Chitra Tirunal Institute for Medical Sciences and Technology, Trivandrum, India).

### 2.2. Method of GTR Membrane Fabrication

Membranes were fabricated by the electrospinning technique using PCL (1 g) and blending the PCL with different weight concentrations of nano-Hydroxyapatite and Bioactive glass. The weight concentrations of both nHA and BG used was 2 wt% (0.02 g), 5 wt% (0.05 g), 10 wt% (0.1 g), and 15 wt% (0.15 g). The initial step was weighing 1 g of PCL followed by weighing of filler either nHAP (nano Hydroxy Apatite) or BG with appropriate wt% concentration; i.e., 2 wt% of either nHAP/BG is added to 1 g PCL and transferred to a glass tube. The next step was the addition of solvents i.e., dichloromethane and dimethylformamide in the ratio of 8:2 mL, respectively, for dissolving the solutes. To this a magnetic pellet was added which acts as a stirrer and placed over an automatic stirring machine. After 3 h of stirring, a clear homogenous solution was obtained.

**Figure 2:**
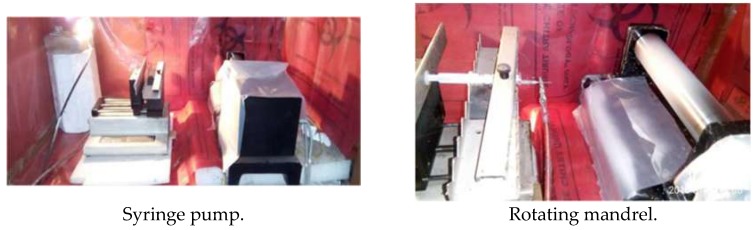

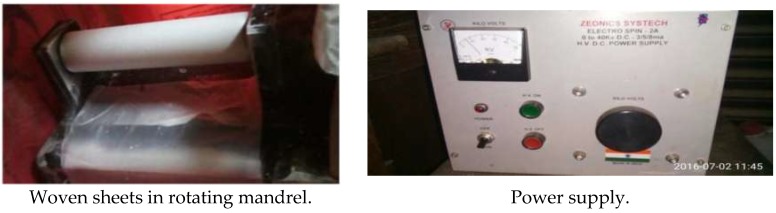
Electrospinning setup.

**Figure 3:**
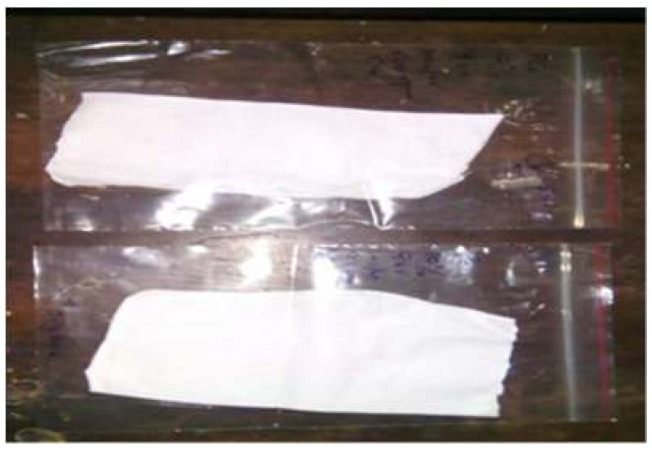
Electrospun sheets.

Spinning was performed at predetermined conditions of 10% solution concentration, applied potential of 12 Kv with a feed rate of 1 mL/h. The desired solutions were loaded into a 10 mL syringe, the opening end of which was connected to a 21-gauge stainless steel needle that was used as the nozzle. A mandrel rotating at 500 rpm was used as the collector and was placed at a distance of 15 cm from the needle tip. A Gamma High-Voltage Research power supply was used to generate a high DC potential. A Scientific syringe pump was used to control the feed rate of the polymer solution. The collection time for the above process was fixed at about 10 h. In the process of electrospinning, a high electrostatic voltage (12 kv) is imposed on a drop of solution held by its surface tension at the end of a needle tip, leading to distortion of the liquid into a conical jet (known as the Taylor cone), ejecting fibers. Once the voltage exceeds a critical value, the electrostatic force overcomes the solution surface tension, and a stable liquid jet is ejected from the cone tip. The solvent evaporates as the jet travels through the air, leaving behind ultra-fine fibers with a high surface-to-volume ratio collected on an electrically-grounded target. After the process, the electrospun fibers in the form of a sheet were obtained (Figure 2 and Figure 3).

A total of 9 samples divided in to three groups were fabricated using the electrospinning technique i.e., an electrospun sample of PCL alone which acts as control group, 4 samples of PCL blended with different concentrations of HA (1 g PCL with 0.02 g, 0.05 g, 0.1 g, 0.15 g) which is considered as a second group, and 4 samples of PCL blended with different concentrations of BG (1 g PCL with 0.02 g, 0.05 g, 0.1g, 0.15 g) as a third group. All these nano-fibrous sheets were dried in a vacuum oven at 40 °C for about 48 h to remove the residual solvent.

### 2.3. Scanning Electron Microscopy (SEM) of Electrospun Scaffolds

**Figure 4:**
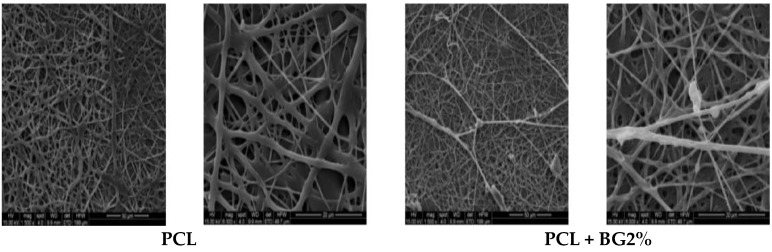

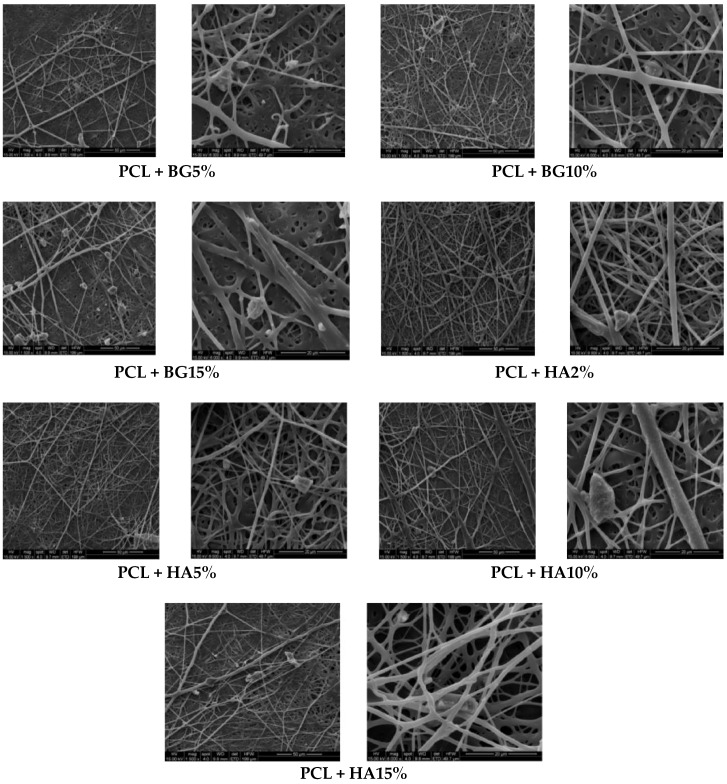
SEM images of fabricated guided tissue regeneration (GTR) membranes at 1500× and 6000× magnifications.

The 3D morphology of the electrospun GTR membranes was observed by scanning electron microscopes (Hitachi-model-S-2400, JEOL, JSM-6390, model 7582, Hitachi, Hokkaido, Japan). The samples were sputter coated with gold palladium and imaged in order to study the fiber morphology. Average fiber diameter, average pore diameter, percentage pore distribution and fiber distribution were measured using Image J software at 1500× and 6000× magnifications (Figure 4).

### 2.4. Mechanical Characterization of Scaffolds

**Figure 5:**
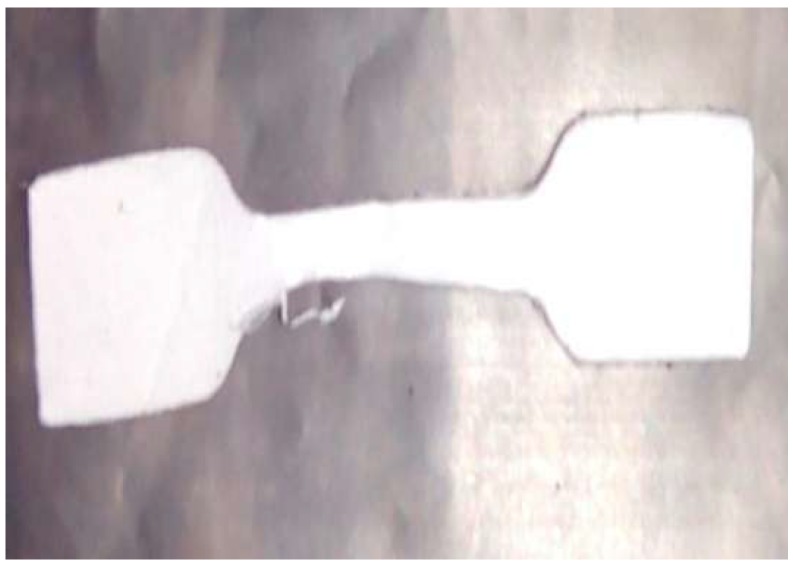
Dumbbell shaped specimen.

The static mechanical properties were determined with a universal testing machine (Instron 3345, single column, Instron, Buckinghamshire, UK) with the use of a 100 N load cell under a crosshead speed of 10 mm/min (Gauge length 20 mm) at ambient conditions. Dumbbell shaped specimens as per ISO standard ISO 527-2 Type 5B (Instron, Buckinghamshire, UK) were used (Figure 5). At least 5 sets of specimens were tested for each type of electrospun fibrous GTR membranes to evaluate tensile strength, Young’s modulus, and elongation at break.

### 2.5. Membrane Thickness

Average thickness was measured using a thickness gauge. The average thickness of the membranes at five random positions was adopted as the mean thickness of the membrane.

### 2.6. In Vitro Hydrolytic Degradation Studies (Hydroscopic Studies)

In vitro hydrolytic degradation behavior of GTR membranes in phosphate buffer solution (PBS) at 37 °C was monitored by observing the changes in weight, morphology, and mechanical properties of scaffolds after different time periods (14 days and 28 days). For weight loss studies, circular samples with 20 mm diameter were incubated in a closed bottle containing 30 mL PBS with a pH of 7.4 at 37 °C. The initial weight (Wi) of the membrane before incubating in PBS was measured and the membranes were retrieved after 28 days. The retrieved membranes were washed with deionized water, dried in a vacuum oven and weighed (Wf) until constant weight was attained. The percentage weight loss was estimated using the equation.

Gravimetric weight loss (%) = (Wi − Wf)/Wi × 100.(1)

For mechanical property evaluation, dumbbell specimens as per ISO standard ISO 527-2 Type 5B were used and the mechanical performance of the membranes after incubating in PBS (pH 7.4) at 37 °C for different time period (14 days and 28 days) was evaluated using a universal testing machine (Instron 3345, single column, Instron, Buckinghamshire, UK).

### 2.7. Chemical Characterization of Scaffolds Using FTIR

The presence of nHAP and BG on PCL fibers was analyzed using ATR-FTIR in a range of 400 cm^−1^ to 4000 cm^−1^ in a non-inert atmosphere (AIM–8800 Shimadzu Corporation, Kyoto, Japan).

### 2.8. Thermal Analysis

Thermogravimetric analysis (TGA) was completed with a Shimadzu TGA-50 (Shimadzu Corporation, Kyoto, Japan). Samples were placed in the balance system and heated from room temperature to 900 °C at a heating rate of 10 °C min^−1^ under a nitrogen atmosphere.

### 2.9. Evaluation of the Toxicity of Material Extracts by MTT Assay

**Figure 6:**
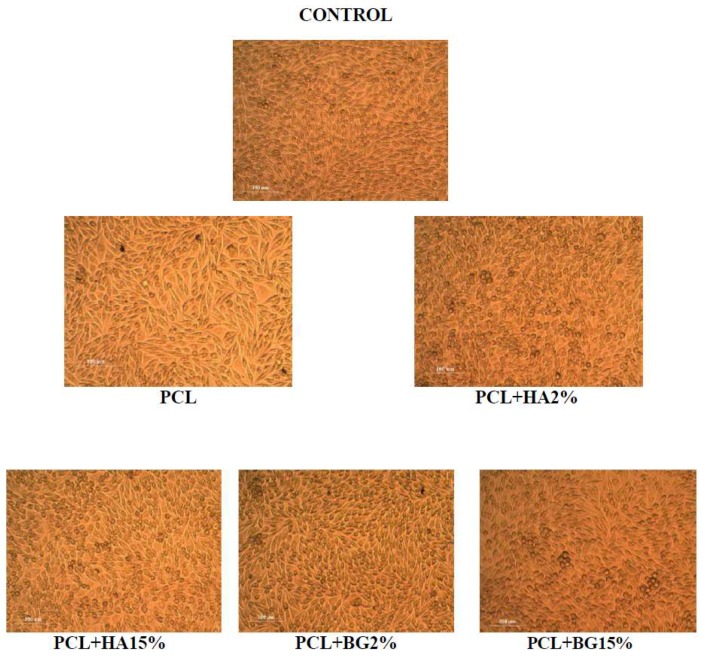
MTT Assay—24 h With L-929 Mouse Fibroblasts.

The cytotoxicity of materials extracts was evaluated as per ISO10993-5 on L-929 mouse fibroblast cell culture. The extract of the material was prepared with DMEM medium by incubating for 48 h. The above medium was used to grow L929 cells for 24 h under standard conditions. The percentage of the surviving fibroblast cells were quantified by the MTT assay and the morphological changes of the cells were monitored by phase contrast microscopy. The optical density (OD) was measured spectro-photometrically at 540 nm. Cells treated with MTT solution without sample were used as control. The % viability was calculated as follows (Figure 6).

Percentage viability = OD of Test/OD of Control × 100(2)

### 2.10. Direct Contact Method

**Figure 7:**
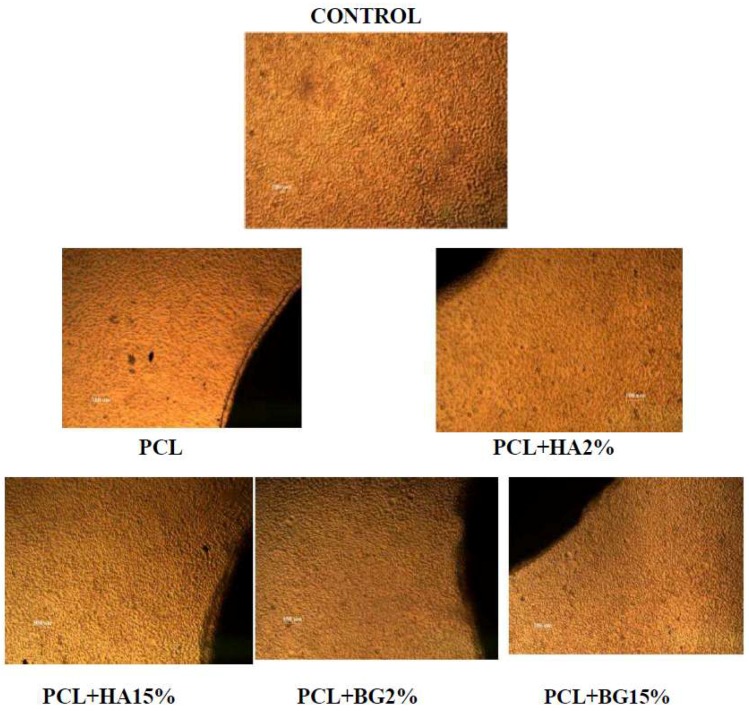
Direct Contact—24 h with L-929 Mouse Fibroblasts.

The cytotoxicity of materials under direct contact with a cell was determined by direct contact assay. L929 fibroblast cells (1 × 10^4^ cells/m) were seeded on to a 24 well plate (BD Falcon, Swedesboro, NJ, USA) and allowed to proliferate for 24 h to form a sub-confluent layer. Then, the material (1 cm diameter) was placed over the monolayer and allowed to proliferate for 24 h in a CO_2_ incubator. After the incubation, cells were evaluated for changes in morphology with respect to a control (cells grown without materials) under inverted phase contrast microscope (Olympus CKX41, Olympus, Tokyo, Japan) attached with an imaging camera. The images were captured using imaging software Optika vision-pro (Figure 7).

## 3. Statistical Analysis

The data were analyzed by Statistical Package for Social Sciences (SPSS 16.0). The data were tested for normality. One-way ANOVA (Post hoc) followed by Dunnet *t* test were applied to find statistical significance between the groups. Chi square test was applied to find the statistical significance within the groups (*p*-value less than 0.05 was considered statistically significant at 95 percent confidence interval).

## 4. Results

**Figure 8:**
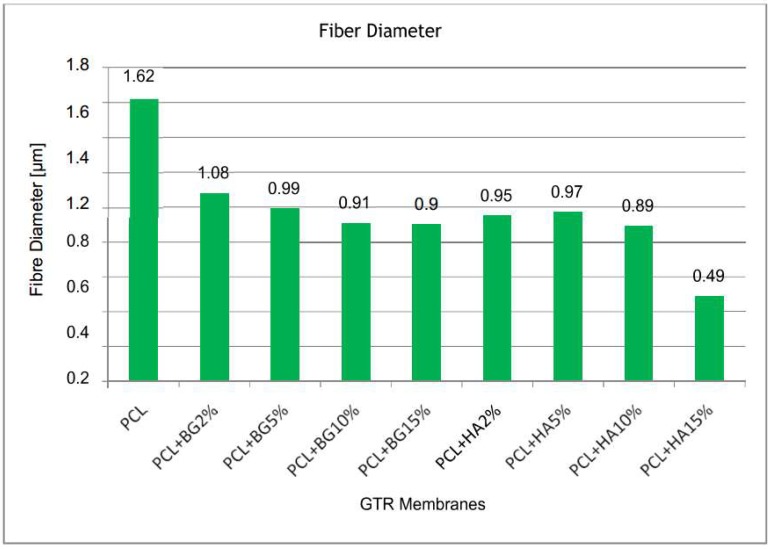
Comparison of Mean Fiber diameter between the groups.

**Figure 9:**
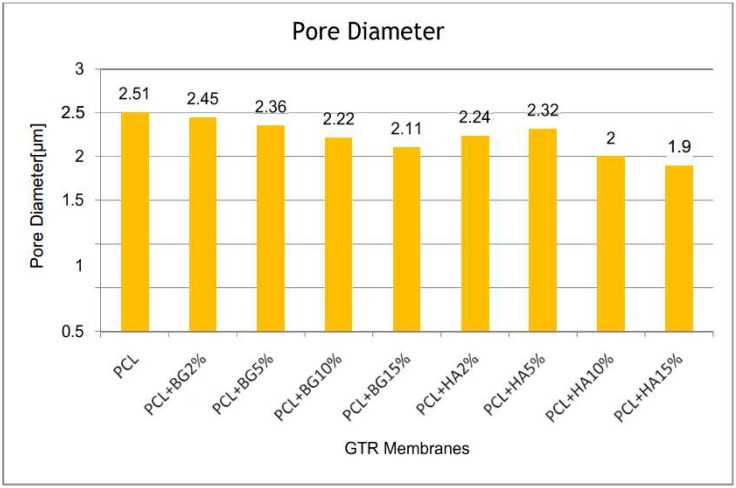
Comparison of Mean Pore diameter between the groups.

Table 1 shows a comparison of mean fiber diameter and pore diameter between the groups obtained from SEM analysis. The magnification used were 1500× and 6000× for all the fabricate membranes. From this table it is evident that PCL membranes showed higher fiber diameter (1.62 ± 0.40) when compared with the fiber diameters of other membranes, and was statistically significant. Among the PCL + BG group of membranes, PCL + BG2% (1.08 ± 0.16) showed higher fiber diameter. Among the PCL + HA group of membranes, PCL + HA2% showed higher fiber diameter. Statistically significant higher pore diameter was exhibited by the PCL membrane (2.51 ± 0.80 µm) when compared with PCL + HA15% (1.90 ± 0.67). Significant difference in pore diameter was shown by all the membranes only when compared with PCL + HA15%. Overall, there was decrease in fiber diameter and pore diameter with increasing different weight concentrations of filler particles (Figure 8 and Figure 9 respectively).

**Figure 10:**
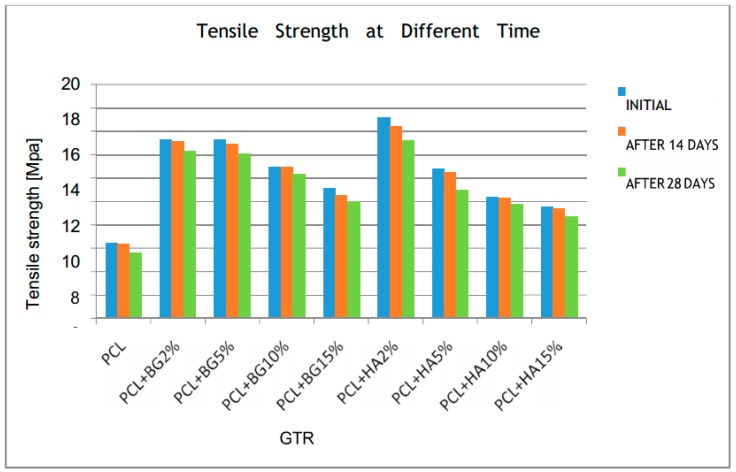
Comparison of mean tensile strength within the groups at different time periods.

Table 2, Table 3, Table 4, Table 5 and Table 6 show the comparison of mean tensile strength (Figure 10), elongation at break, Young’s modulus, weight loss and thickness of fabricated membranes at different time periods, respectively.

On comparing the initial tensile strength with that of tensile strength at 28 days, all the membranes exhibited statistically significant reduction. Among the composite membranes, PCL + HA2% had a higher significance (*p*-value < 0.01) and overall it is evident that tensile strength decreases with the increase in different weight concentrations of filler particles.

**Figure 11:**
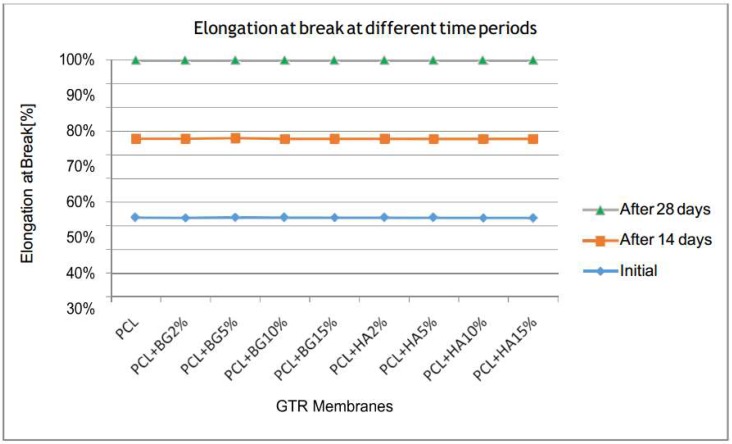
Comparison of mean elongation at break within the groups at different time periods.

On comparison of mean elongation at break within the groups at different time periods, all the membranes exhibited a mean reduction in elongation at break after 14 days and 28 days when compared with the initial value. But the reduction showed by the PCL membrane was statistically significant with a *p*-value of 0.05 (Figure 11).

**Figure 12:**
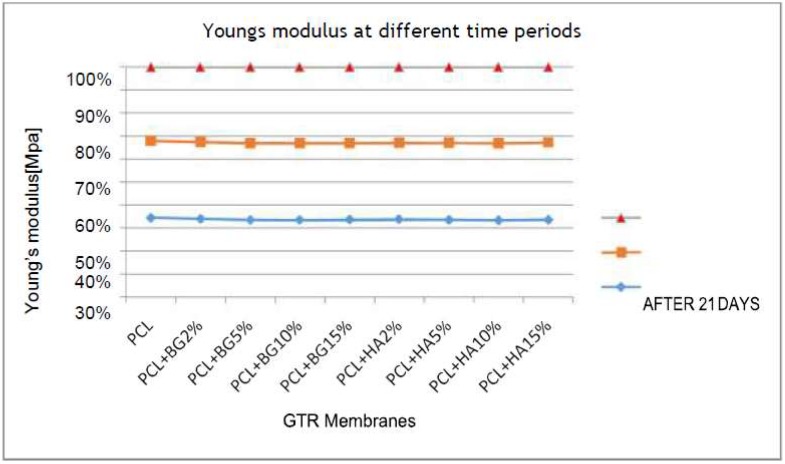
Comparison of mean Young’s modulus within the groups at different time periods.

On comparing the mean Young’s modulus within the groups at different time periods, all of the membranes showed reduction in Young’s modulus when comparing the initial with Young’s modulus after 28 days of degradation. But statistically significant degradation was observed only in PCL + BG2% membrane i.e., from 55.46 ± 8.06 MPa to 53.18 ± 14.25 MPa (*p*-value < 0.05) (Figure 12).

**Figure 13:**
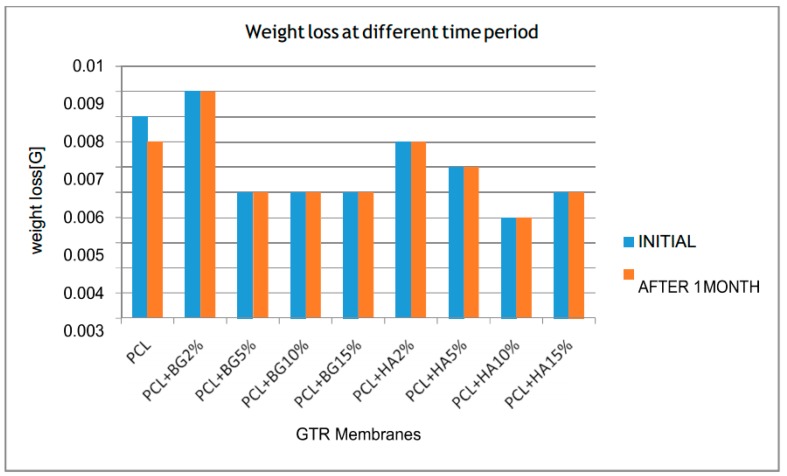
Comparison of mean weight loss within the groups at different time periods.

On comparing mean weight loss (Hydroscopic analysis) within the groups at different time periods, none of the membranes exhibited significant weight loss. Initial weight of the membrane remains constant after one month for all the membranes except for PCL (0.008 ± 0.02 g reduced to 0.007 ± 0.01 g (Figure 13).

**Figure 14:**
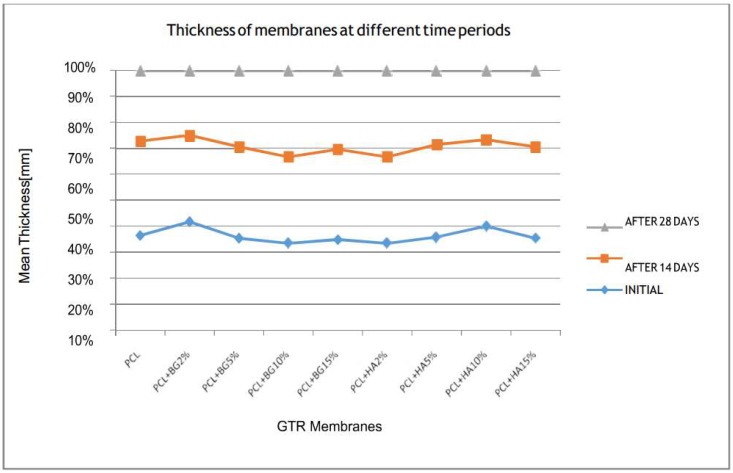
Comparison of mean thickness within the groups at different time periods.

On comparing the mean thickness of membranes within the groups at different time periods, the majority of the membranes showed a reduced thickness after 28 days when compared with the initial thickness and was not statistically significant (*p*-value > 0.05) (Figure 14).

**Figure 15:**
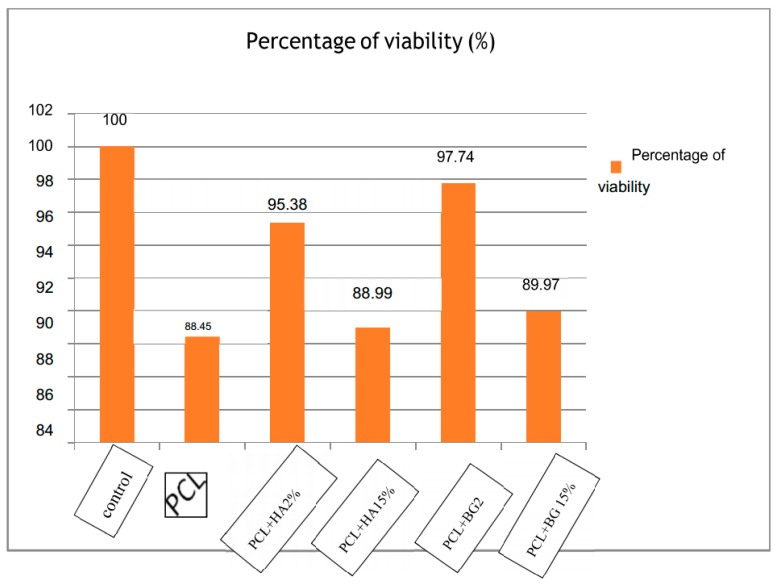
Comparison of percentage viability of cells between the groups at 24 h.

The PCL membrane exhibited 88.45% cell viability and was statistically significant when compared to control and also with PCL + HA2%, PCL + BG2%, 15%. Among the composite membranes, PCL + BG2% (97.74%) showed a higher percentage of viability after 24 h and PCL + HA15% (88.99%]) showed the least percentage of viable cells and was statistically significant. So on comparison, the composite membranes exhibited enhanced cell viability when compared with the PCL membrane alone (Figure 15).

## 5. Discussion

In the present study, three different GTR membranes were made, grouped as PCL; PCL + BG; and PCL + HA. PCL + BG and PCL + HA consisted of membranes fabricated with four different weight concentrations of BG/nHAP, i.e., PCL + BG2% PCL + BG5%; PCL + BG10%; and PCL + BG15%. Similarly, the PCL + HA group also consisted of PCL + HA2%; PCL + HA5%; PCL + HA10% and PCL + HA15%. Morphological evaluation of the fabricated membranes was done using SEM. There was a statistically significant difference in fiber and pore diameter for the PCL membrane and also for PCL + HA2%, PCL + HA5% PCL + HA10%, PCL + HA15%, and PCL + BG2%, PCL + BG5%, PCL + BG10%, PCL + BG15% GTR membranes (Table 1). For PCL, non-uniform fibers with an average diameter of 1.62 μm and pore diameter of 2.51 μm were obtained. As evident from the SEM micrograph, blending PCL with different weight concentrations of BG and nHAP resulted in a reduction of fiber diameters and pore diameter. Among the PCL blended with BG and nHAP membranes, PCL + BG2% showed statistically significant increased fiber diameter (1.08 ± 0.16 µm) and pore diameter (2.45 ± 0.92 µm). The fiber diameter and pore diameter was much less for PCL + HA15% (0.49 ± 0.58 µm and 1.90 ± 0.67 µm, respectively). Overall the fibers showed decreased fiber and pore diameters with the addition of different weight concentrations of filler particles when compared to PCL fibers alone. The findings of the present study were consistent with the study done by Remya et al. in 2013 where the fabricated PCL membrane had a fiber diameter of 1.54 µm.

The electrospinning technique is governed by various processing parameters such as solution viscosity, applied potential, flow rate, tip to collector distance, solvent nature, needle diameter, and various ambient parameters. In the present study, spinning was done at a predetermined optimized condition to get bead free fibers. It is observed that blending PCL with different concentrations of bioactive materials resulted in smooth fibers with reduced fiber diameter. This reduction in fiber diameter can be attributed to the difference in solution viscosity i.e., when viscosity decreases conductivity increases and this conductivity is inversely proportional to fiber diameter. A reduction in fiber diameter was observed with the incorporation of nHAP particles and this can be attributed to the presence of calcium and phosphate ions in nHAP which provide higher conductivity [18].

Pore diameter and fiber diameter plays a vital role in the biological performance of scaffold as it determines both cell–cell as well as cell–membrane interaction. High porosity, adequate pore size and interconnected pore network are essential criteria for tissue engineering as it enables better cell infiltration and vascularization [18,19]. The reduction in pore size occurs as more layers of fibers have the possibility to overlap with each other, especially when the fiber diameter is smaller, which ultimately results in smaller pore diameter. The pore size distribution lies in a range below 300 µm for all the scaffolds. The preferable pore size for osteoblast cells ranges from 200 to 400 µm for encouraging migration, attachment, and proliferation. However, for electrospun matrices, the pores formed are much smaller than the normal cell size of a few to tens of micrometers. Pores in an electrospun structure are formed by the randomly oriented fibers lying loosely upon each other. Cells can migrate through pores by their amoeboid movement and can push surrounding fibers aside to expand the hole. This dynamic architecture of fibers allows cells to adjust according to pore size and grow into nanofiber matrices [18].

In this study, PCL + BG2% showed statistically significant increased fiber diameter (1.08 ± 0.16 μm; *p* < 0.03) and pore diameter (2.45 ± 0.92 μm; *p* < 0.04) when compared to other membranes.

The mechanical properties of the fabricated GTR membranes observed in this study included tensile strength, Young’s Modulus, and elongation at break. All the membranes exhibited statistically significant difference in mechanical properties between and within the groups (Table 7). Among the different GTR membranes, higher tensile strength was exhibited by PCL + HA2% (17.22 ± 2.80 MPa) and PCL + HA15% showed the least tensile strength (9.55 ± 1.04). The superiority in their tensile strength was maintained by the membranes initially, after 14 days, and one month after degradation in PBS, and was statistically significant. These values that we obtained in our study are higher than the values of tensile strength obtained by Yang et al. [7] and Yunzhu et al. [20]. In case of Young’s modulus, PCL + HA2% exhibited the highest Young’s modulus (57.16 ± 7.39) and the least was exhibited by PCL (28.01 ± 5.98 MPa) initially, after 14 days, and one month after degradation in PBS; this was consistent with the studies done by Yang et al. [7] where Young’s modulus was about 19.46 MPa for PCL + HA composite membranes. In surgery, GTR membranes are tightly fixed by biodegradable pins, medical glue, or sutures preventing them from sagging into bone defects. Our mechanical test showed that the addition of nHAP increased the mechanical properties of the PCL membrane. Among the different membranes that we tested in this study, the PCL + HA2% and PCL + BG2% showed the highest tensile strength, and Young’s modulus. With an increasing nHAP content, the higher concentrations of PCL + BG and PCL + HA (5%, 10%, 15%) showed weakened properties compared with PCL + HA2% but remained stronger and tougher than the pure PCL membrane. This mechanical reinforcement effect can be attributed to an additional energy-dissipating mechanism introduced by the nanoparticles in PCL. Recent molecular dynamics studies suggested that this additional dissipative mechanism is a result of the mobility of the nanoparticles [7]. During the deformation process, the nanoparticles orient and align under tensile stress, creating temporary cross-links between polymer chains, thereby creating a local region of enhanced strength. However, when the size of the nHAP/BG stacks is increased, they become less mobile. Therefore, the ability of the nHAP/BG to dissipate energy is also reduced, resulting in almost no improvement in the toughness of the material [7]. Findings from our study were consistent with the study done by Remya et al. in 2013 which states that membranes with smaller fiber diameters will provide higher overall relative bonded areas between fibers due to the increased surface area, bonding density, and better distribution of bonds [18].

Considering elongation at break or elasticity of the fabricated GTR membranes, the highest elongation at break was exhibited by PCL (259.25 ± 6.95%) and least was exhibited by PCL + HA2% (127.80 ± 7.38) initially, after 14 days, and one month after degradation in PBS and was statistically significant. The elasticity of a material is inversely proportional to the strength; i.e., PCL + HA2% had increased tensile strength so the membrane exhibited least elasticity/elongation at break. All the membranes maintained its initial superiority in mechanical properties even after one month of in vitro degradation in PBS.

To the best of our knowledge, no studies have evaluated the thickness and weight of the electrospun GTR membrane before and after in vitro degradation. Among the different GTR membranes, mean higher weight appeared in PCL + BG2% (0.009 ± 0.01 g) and lower weight by PCL + HA10% (0.004 ± 0.01 g) and the differences between them was statistically significant. We speculate that this discrepancy is be due to a difference in dispersion of particles. In the case of thickness, the highest thickness appeared in PCL + BG15% (0.08 ± 0.02 mm) and least thickness appeared in PCL [0.04 ± 0.01 mm] which was statistically significant, which is attributed to the increased weight concentrations of bioactive materials.

Evaluating the chemical stability of the fabricated membranes by FTIR, the spectra revealed that PCL exhibited its characteristic absorption bands for C=O stretching vibrations from ester bond at 1732 cm^−1^, CH_2_ stretching vibrations at 2973 cm^−1^ and 2861 cm^−1^, and that of C–O vibrations from ether groups at 1240 cm^−1^, respectively. Similarly the FTIR spectrum of HA showed characteristic functional groups of phosphate and hydroxyl moieties. Phosphate peaks were observed d70 cm^−1^ and 1050 cm^−1^ (572.4 cm^−1^, 601.2 cm^−1^, 987.2 cm^−1^, 1053 cm^−1^). The characteristic hydroxyl peak was observed at 3570.6 cm^−1^. The FTIR spectrum of BG showed characteristic functional groups of silicate absorption bands assigned to the peaks 1055, 908, and 482 cm^−1^, respectively (asymmetric stretching mode, symmetric stretching vibration, and rocking vibration of Si–O–S).

The inference from FTIR analysis is that nHA and BG were successfully incorporated in PCL membrane without any undesirable chemical reaction or loss of chemical structure of all these three components indicated by the appearance of signature peaks of PCL, nHA and BG in PCL + nHA and PCLBG composite membranes. These findings of FTIR analysis were consistent with the study done by Yunzhu et al. [20].

The presence of BG and nHA in PCL scaffolds was analyzed using thermogravimetric analysis. For PCL, complete charring occurred at 420 °C, whereas for the composite membranes, even after 420 °C the weight percentage of filler remaining was of 27% for PCL + nHA2% and 29% for PCL + nHA15% composites, and 2.2% for PCL + BG2% and 7.7% for PCL + BG15% composite membranes. This shows that the composite membranes exhibited high thermal stability. The study done by Neethu et al. states that complete charring of the PCL occurred at 428 °C [21]. This finding was not consistent with the present study. The reason for this might be the difference in molecular weight of the PCL they used in that study (Mw 70,000–90,000) [21].

Apart from favorable physicochemical and mechanical properties, the most important requirement for a biomaterial is its biocompatibility in a specific environment, together with the non-cytotoxicity of its degradation products [21]. As a preliminary step towards the evaluation of cyto-compatibility of the scaffold, MTT assay was performed and the result reveals the non-cytotoxic nature of the tested membranes i.e., PCL, PCL + BG2%, PCL + BG15%, and PCL + HA2%, PCL + HA15%. The percentage viability of cells on the membrane determine the suitability of the material for the intended application. In this study, the potential of fabricated membranes for tissue engineering applications was evaluated by in vitro cell culture studies using L929 Mouse fibroblast cell lines. Quantitative and qualitative assays proved that the fabricated membranes are nontoxic. The cells maintained their characteristic spindle morphology on all the GTR membranes after 24 h. The percentage cell viability was higher in PCL + BG2% (97.74, *p* < 0.04) after 24 h and was statistically significant when compared with the rest of the GTR membranes (Table 8).

Ogawa et al. in 2016 [22] stated that nano-modification of biomaterials might increase the surface area and adsorption of signaling molecules. In our study, the PCL + BG2% exhibited higher fiber diameter (1.08 ± 0.16 μm). As a result of this, the membrane showed an increased surface area which results in higher percentage of viable cells [23]. This observation confirms the bioactivity of the fabricated membranes and its usefulness as a Guided tissue regeneration membrane for proliferation and differentiation into specific cell lineages.

Even though the results of present study showed that the electrospun GTR membranes possessed adequate in vitro properties and supported cell attachment, there are certain limitations of the study. The cell part of the study here was of limited size and in the future the membranes should be completely tested using different cell types, e.g., gingival fibroblasts, periodontal ligament fibroblasts, and osteoblast-like cells in terms of cell attachment, proliferation, migration, penetration, differentiation, and mineralization. Another limitation is that the present study did not use any surfactant in order to achieve a stable and uniform nHAP/BG suspension in the polymer solutions.

## 6. Conclusions

The optimal resorbable membrane for GTR or GBR has to be strong, able to stimulate bone formation, and promote osteoblast-like cell proliferation and differentiation. In the present study, composite nanofibrous membranes based on nHAP/BG and PCL were prepared by electrospinning. Physiochemical, mechanical, and in vitro characterization showed that the composite membrane fulfilled all aforementioned requirements. However, the membrane with a high nHAP/BG loading density was weaker than the one with low nHAP/BG loading density. On the basis of our results, we conclude that the composite nanofibrous membrane prepared by electrospinning is suitable for use as a GTR membrane and it is a useful prototype for further development of a final membrane for clinical use.

## Figures and Tables

**Table 1:** Fiber diameter and pore diameter of the fabricated guided tissue regeneration (GTR) membranes.

Groups	Fiber Diameter (µm) (MEAN ± SD)	Pore Diameter (µm) (MEAN ± SD)
**PCL**	1.62 ± 0.40	2.51 ± 0.80
**PCL + BG2%**	1.08 ± 0.16	2.45 ± 0.92
**PCL + BG5%**	0.99 ± 0.24	2.36 ± 1.19
**PCL + BG10%**	0.91 ± 0.23	2.22 ± 0.79
**PCL + BG15%**	0.90 ± 0.21	2.11 ± 0.60
**PCL + HA2%**	0.95 ± 0.22	2.24 ± 0.82
**PCL + HA5%**	0.97 ± 0.33	2.32 ± 0.70
**PCL + HA10%**	0.89 ± 0.26	2.00 ± 0.72
**PCL + HA15%**	0.49 ± 0.58	1.90 ± 0.67

**Table 2:** It shows the comparison of mean Tensile Strength at different time periods.

Groups	Tensile Strength at Initial	Tensile Strength at 14 Days	Tensile Strength at 28 Days	*p*-Value
	(MEAN ± SD) (MPa)	(MEAN ± SD) (MPa)	(MEAN ± SD) (MPa)	
**PCL**	6.50 ± 0.38	6.40 ± 0.07	5.67 ± 0.58 *	0.04
**PCL + BG2%**	15.35 ± 1.91	15.14 ± 0.99	14.30 ± 4.68 *	0.03
**PCL + BG5%**	15.35 ± 0.89	14.92 ± 1.60	14.07 ± 1.00 *	0.04
**PCL + BG10%**	13.00 ± 0.01	13.00 ± 5.05	12.37 ± 2.82 *	0.03
**PCL + BG15%**	11.10 ± 3.54	10.56 ± 2.42	9.99 ± 4.58 *	0.03
**PCL + HA2%**	17.22 ± 2.80	16.46 ± 0.12	15.28 ± 1.88 *	0.01
**PCL + HA5%**	12.86 ± 0.65	12.55 ± 0.75	11.03 ± 5.17 *	0.03
**PCL + HA10%**	10.42 ± 0.21	10.34 ± 1.28	9.83 ± 3.84 *	0.04
**PCL + HA15%**	9.55 ± 1.04	9.39 ± 1.48	8.75 ± 2.89 *	0.04

*, *p* < 0.05 considered statistically significant compared between the groups.

**Table 3:** It shows the comparison of mean elongation at break at different time periods.

Groups	Initial Elongation at Break (%)	Elongation at Break after 14 Days (%)	Elongation at Break after 28 Days (%)	*p*-Value
	(MEAN ± SD)	(MEAN ± SD)	(MEAN ± SD)	
**PCL**	259.25 ± 6.95	258.00 ± 67.08	257.12 ± 62.86 *	0.05
**PCL +BG2%**	153.08 ± 10.66	152.86 ± 10.52	152.35 ± 4.78	1.56
**PCL +BG5%**	167.00 ± 18.09	166.63 ± 17.38	163.91 ± 14.85	0.86
**PCL +BG10%**	171.94 ± 27.83	171.14 ± 27.83	170.96 ± 74.81	1.78
**PCL + BG15%**	182.74 ± 35.30	182.21 ± 20.07	181.30 ± 1.44	1.34
**PCL + HA2%**	127.80 ± 7.38	127.60 ± 8.48	126.82 ± 10.54	1.18
**PCL + HA5%**	179.30 ± 23.43	178.49 ± 22.33	178.22 ± 10.02	1.34
**PCL + HA10%**	207.33 ± 26.48	207.33 ± 31.27	206.72 ± 33.35	0.98
**PCL + HA15%**	211.34 ± 50.00	211.12 ± 85.70	210.83 ± 26.05	1.34

*, *p* < 0.05 considered statistically significant compared between the groups.

**Table 4:** It shows comparison of mean Young’s modulus at different time periods.

Groups	Young’s Modulus at Initial (MPa)	Young’s Modulus after 14 Days (MPa)	Young’s Modulus after 28 Days (MPa)	*p*-Value
	(MEAN ± SD)	(MEAN ± SD)	(MEAN ± SD)	
**PCL**	28.01 ± 5.98	27.07 ± 2.13	26.07 ± 2.08	1.89
**PCL + BG2%**	55.46 ± 8.06	54.22 ± 8.61	53.18 ± 14.25 *	0.04
**PCL + BG5%**	53.28 ± 6.76	53.10 ± 0.92	52.81 ± 8.43	1.7
**PCL + BG10%**	51.34 ± 8.69	51.29 ± 0.87	50.87 ± 15.46	1.45
**PCL + BG15%**	31.74 ± 1.48	31.26 ± 1.20	31.05 ± 10.03	1.34
**PCL + HA2%**	57.16 ± 7.39	56.29 ± 7.84	55.72 ± 11.16 *	0.04
**PCL + HA5%**	37.70 ± 2.53	37.54 ± 0.88	36.94 ± 12.36	1.45
**PCL + HA10%**	31.13 ± 2.03	31.07 ± 0.99	30.96 ± 1.98	1.84
**PCL + HA15%**	31.07 ± 3.39	31.06 ± 1.40	30.30 ± 17.64	1.56

*, *p* < 0.05 considered statistically significant compared between the groups.

**Table 5:** It shows hydroscopic analysis within the groups at different time periods.

Groups	Initial Weight (mm)	Weight after One Month (mm)	*p*-Value
	(MEANs ± SD)	(MEAN ± SD)	
**PCL**	0.008 ± 0.02	0.007 ± 0.01	0.56
**PCL + BG2%**	0.009 ± 0.02	0.009 ± 0.01	1.78
**PCL + BG5%**	0.005 ± 0.01	0.005 ± 0.01	1.45
**PCL + BG10%**	0.005 ± 0.01	0.005 ± 0.02	1.45
**PCL + BG15%**	0.005 ± 0.01	0.005 ± 0.01	1.45
**PCL + HA2%**	0.007 ± 0.01	0.007 ± 0.01	1.67
**PCL + HA5%**	0.006 ± 0.01	0.006 ± 0.02	1.36
**PCL + HA10%**	0.004 ± 0.01	0.004 ± 0.01	1.78
**PCL + HA15%**	0.005 ± 0.02	0.005 ± 0.01	1.45

*p* < 0.05 considered statistically significant compared between the groups.

**Table 6:** It shows comparison of mean thickness of membranes within the groups at different time periods.

Groups	Initial Thickness (mm)	Thickness at 14 Days (mm)	Thickness at 28 Days (mm)	*p*-Value
	(MEAN ± SD)	(MEAN ± SD)	(MEAN ± SD)	
**PCL**	0.04 ± 0.01	0.04 ± 0.01	0.03 ± 0.01	1.45
**PCL + BG2%**	0.05 ± 0.02	0.04 ± 0.02	0.03 ± 0.02	1.23
**PCL + BG5%**	0.06 ± 0.01	0.06 ± 0.01	0.05 ± 0.01	0.56
**PCL + BG10%**	0.07 ± 0.01	0.07 ± 0.01	0.07 ± 0.01	0.92
**PCL + BG15%**	0.08 ± 0.02	0.08 ± 0.02	0.07 ± 0.01	1.34
**PCL + HA2%**	0.04 ± 0.01	0.04 ± 0.01	0.04 ± 0.01	1.23
**PCL + HA5%**	0.05 ± 0.01	0.05 ± 0.01	0.04 ± 0.01	0.89
**PCL + HA10%**	0.06 ± 0.01	0.05 ± 0.01	0.04 ± 0.01	1.56
**PCL + HA15%**	0.06 ± 0.01	0.06 ± 0.01	0.05 ± 0.04	1.67

*p* < 0.05 considered statistically significant compared between the groups.

**Table 7:** Mechanical properties of fabricated guided tissue regeneration (GTR) membranes at different time periods.

Groups	Tensile Strength (MPa) (MEAN ± SD)			Young’s Modulus (MPa) (MEAN ± SD)			Elongation at Break (%) (MEAN ± SD)			Weight Loss (g)		Thickness (mm)	
	Initial	14 Days	28 Days	Initial	14 Days	28 Days	Initial	14 Days	28 Days	Initial	28 Days	Initial	28 Days
**PCL**	6.50 ± 0.38	6.40 ± 0.07	5.67 ± 0.58 *	28.01 ± 5.98	27.07 ± 2.13	26.07 ± 2.08	259.25 ± 6.95	258.00 ± 67.08	257.12 ± 62.86 *	0.008 ± 0.02	0.007 ± 0.01	0.04 ± 0.01	0.03 ± 0.01
**PCL + BG2%**	15.35 ± 1.91	15.14 ± 0.99	14.30 ± 4.68 *	55.46 ± 8.06	54.22 ± 8.61	53.18 ± 14.25 *	153.08 ± 10.66	152.86 ± 10.52	152.35 ± 4.78	0.009 ± 0.02	0.009 ± 0.01	0.05 ± 0.02	0.03 ± 0.02
**PCL + BG5%**	15.35 ± 0.89	14.92 ± 1.60	14.07 ± 1.00 *	53.28 ± 6.76	53.10 ± 0.92	52.81 ± 8.43	167.00 ± 18.09	166.63 ± 17.38	163.91 ± 14.85	0.005 ± 0.01	0.005 ± 0.01	0.06 ± 0.01	0.05 ± 0.01
**PCL + BG10%**	13.00 ± 0.01	13.00 ± 5.05	12.37 ± 2.82 *	51.34 ± 8.69	51.29 ± 0.87	50.87 ± 15.46	171.94 ± 27.83	171.14 ± 27.83	170.96 ± 74.81	0.005 ± 0.01	0.005 ± 0.02	0.07 ± 0.01	0.07 ± 0.01
**PCL + BG15%**	11.10 ± 3.54	10.56 ± 2.42	9.99 ± 4.58 *	31.74 ± 1.48	31.26 ± 1.20	31.05 ± 10.03	182.74 ± 35.30	182.21 ± 20.07	181.30 ± 1.44	0.005 ± 0.01	0.005 ± 0.01	0.08 ± 0.02	0.07 ± 0.01
**PCL + HA2%**	17.22 ± 2.80	16.46 ± 0.12	15.28 ± 1.88 *	57.16 ± 7.39	56.29 ± 7.84	55.72 ± 11.16 *	127.80 ± 7.38	127.60 ± 8.48	126.82 ± 10.54	0.007 ± 0.01	0.007 ± 0.01	0.04 ± 0.01	0.04 ± 0.01
**PCL + HA5%**	12.86 ± 0.65	12.55 ± 0.75	11.03 ± 5.17 *	7.70 ± 2.53	37.54 ± 0.88	36.94 ± 12.36	179.30 ± 23.43	178.49 ± 22.33	178.22 ± 10.02	0.006 ± 0.01	0.006 ± 0.02	0.05 ± 0.01	0.04 ± 0.01
**PCL + HA10%**	10.42 ± 0.21	10.34 ± 1.28	9.83 ± 3.84 *	31.13 ± 2.03	31.07 ± 0.99	30.96 ± 1.98	207.33 ± 26.48	207.33 ± 31.27	206.72 ± 33.35	0.004 ± 0.01	0.004 ± 0.01	0.06 ± 0.01	0.04 ± 0.01
**PCL + HA15%**	9.55 ± 1.04	9.39 ± 1.48	8.75 ± 2.89 *	31.07 ± 3.39	31.06 ± 1.40	30.30 ± 17.64	211.34 ± 50.00	211.12 ± 85.70	210.83 ± 26.05	0.005 ± 0.02	0.005 ± 0.01	0.06 ± 0.01	0.05 ± 0.04

*, *p* < 0.05 considered statistically significant compared between the groups.

**Table 8:** Percentage viability of cells at 24 h.

Groups	Percentage of Viability (%)
**Cell Control**	100
**PCL**	88.45
**PCL + HA2%**	95.38
**PCL + HA15%**	88.99
**PCL + BG2%**	97.74
**PCL + BG15%**	89.97
